# Supplementary material for: GRADE Use in Evidence Syntheses Published in High-Impact-Factor Gynecology and Obstetrics Journals: A Methodological Survey
Source: J Clin Med. 2023 Jan 5;12(2):446. doi: 10.3390/jcm12020446 (PMC9866985; doi:10.3390/jcm12020446)
Supplement: Supplementary file 1 [file jcm-12-00446-s001.zip › jcm-2050266-supplementary.pdf]

Appendix S1:

("Hum Reprod Update"[jour] OR "Am J Obstet Gynecol"[jour] OR "Obstet Gynecol"[jour] OR "Hum Reprod"[jour] OR "Fertil Steril"[jour] OR "Ultrasound Obstet Gynecol"[jour] OR "BJOG"[jour] OR "Gynecol Oncol"[jour] OR "Best Pract Res Clin Obstet Gynaecol"[jour] OR "Breast"[jour]) AND ("Systematic Review"[Publication Type] OR "systematic review"[tiab] OR meta-analysis[Publication Type] OR meta-analysis[tiab])

("Hum Reprod Update"[jour] OR "Am J Obstet Gynecol"[jour] OR "Obstet Gynecol"[jour] OR ""[jour] OR "Hum Reprod"[jour] OR "Fertil Steril"[jour] OR " Ultrasound Obstet Gynecol "[jour] OR " BJOG"[jour] OR " Gynecol Oncol"[jour] OR " Best Pract Res Clin Obstet Gynaecol"[jour] OR " Breast"[jour]) AND ("Systematic Review"[Publication Type] OR "systematic review"[tiab] OR meta-analysis[Publication Type] OR meta-analysis[tiab])

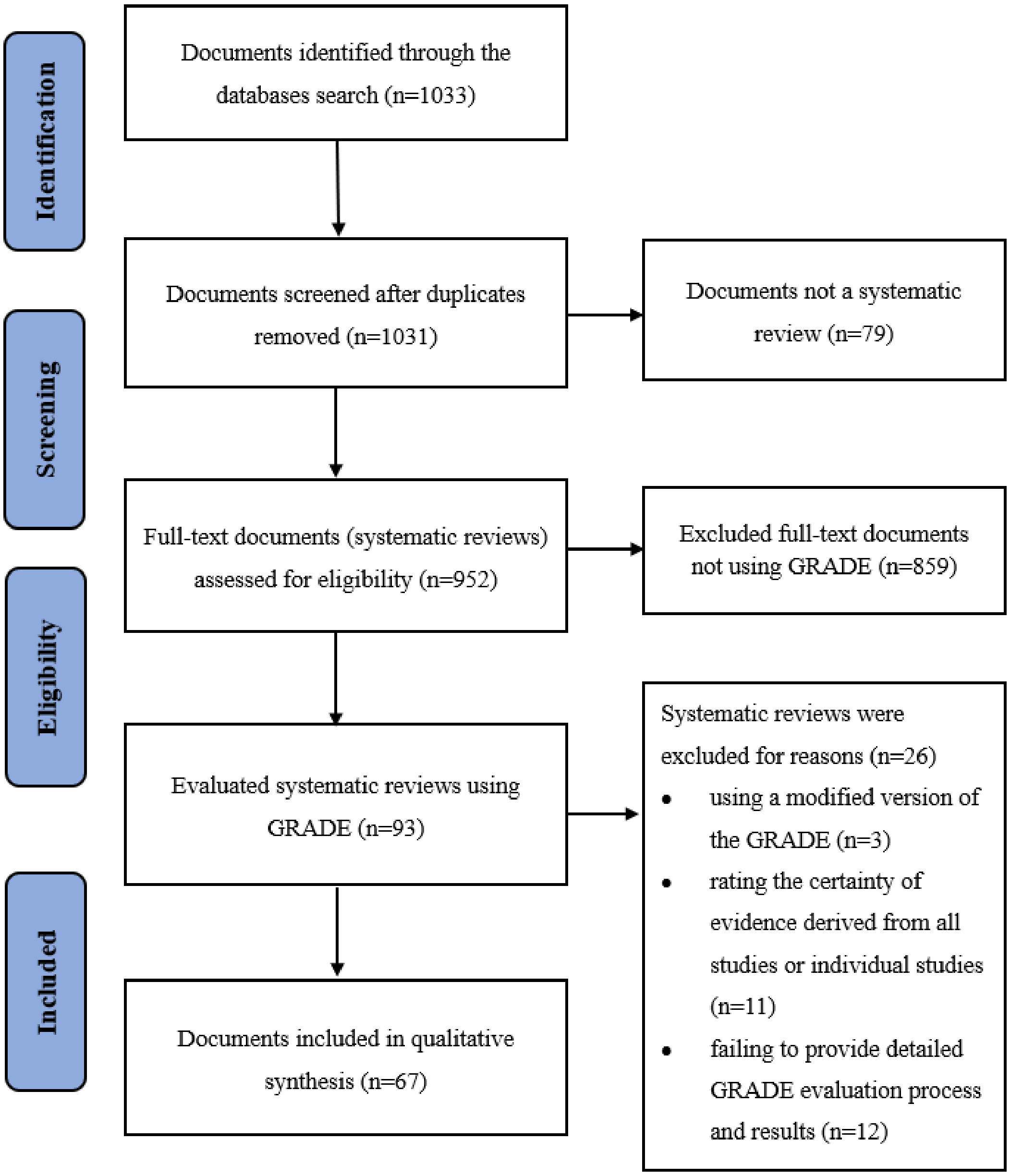

Figure S1: Flow diagram showing study selection process.

Table S1: Overview of SRs that applied GRADE incorrectly* by rating the certainty of evidence for all studies or each individual study.

Author Ref, Year	Number of studies	Type of studies	Number of participants	Intervention/Exposure	Application of GRADE
Bogani et al. 2016 [32]	5	NRS	360	adjuvant chemotherapy	Certainty of evidence of individual studies was assessed (not outcome specific)
Evans et al. 2016 [33]	34	NRS	unclear	Salpingo-oophorectomy	Certainty of evidence of individual studies was assessed (not outcome specific)
Blanton et al. 2017 [34]	24	RCT& NRS	1919	ketorolac, intravenous acetaminophen, anti-convulsants and dexamethasone	Certainty of evidence of individual studies was assessed (not outcome specific)
Gemmell et al. 2017 [35]	39	NRS &RCT	unclear	hormone replacement therapy, hormone replacement therapy	Certainty of evidence of individual studies was assessed (not outcome specific)
Joint et al. 2018 [36]	43	NRS	unclear	gender-affirming hormones	Certainty of evidence of individual studies was assessed (not outcome specific)
Simsek et al. 2018 [37]	28	NRS	1016684	thiopurines	Certainty of evidence of individual studies was assessed (not outcome specific)
Nahshon et al. 2020 [38]	8	NRS	13098	BRCA1/2mutation	Certainty of evidence of individual studies was assessed (not outcome specific)
Åsenius et al. 2020 [39]	135	NRS	2873	spermatozoal DNA methylation	Certainty of evidence of individual studies was assessed (not outcome specific)
Nudelman et al. 2020 [40]	5	RCT&NRS	452	Delayed Cord Clamping	Certainty of evidence of individual studies was assessed (not outcome specific)

Orr et al. 2020 [41]	30	RCT	6,465	Foley catheter and prostaglandins or Foley and oxytocin	Certainty of evidence of individual studies was assessed (not outcome specific)
Storgaard et al. 2017 [42]	35	NRS	unclear	oocyte donation	Certainty of evidence of individual studies was assessed (not outcome specific)

* The statement “incorrect use of GRADE” refers to systematic reviews that assessed only the certainty of evidence derived from all studies or individual studies included in a systematic review, instead of grading the outcome specific certainty of evidence or the body of evidence for a given outcome.

Notes: NRSs: non-randomized studies; RCTs: randomized controlled trials;

Table S2: Overview of the GRADE domains of systematic reviews that rated the outcome-specific certainty of evidence.

**Author, Year**	**Meta-analysis conducted**	**Strength of recommendations**	**Summary of Findings Table**	**Outcome importance reported**	**Downgrading factors**	**Upgrading**
Mowat et al. 2016 [85]	Y	N	Y	N	Imprecision; Inconsistency (Significant heterogeneity with an I2=85%)	Plausible Confounder（Inclusion of gynecologic-oncologists diminishes the volume); Large effect（The magnitude of effect was large)
Wei et al. 2016 [44]	Y	N	Y	N	Risk of bias (poor description of the study design; random sequence generation and allocation concealment); Imprecision (The extent of overlap of confidence intervals is small, and I2 = 93%; The sample size of the studies is less than optimal information size, and the 95% CI includes a mean difference of 0; the 95% CI includes a relative risk of 1.0); Publication bias	-

Rydén et al. 2016 [45]	Y	N	Y	N	Risk of bias; Imprecision; Indirectness; Inconsistency (Downgrading reasons not explained).	-
Nastri et al. 2016 [46]	Y	N	Y	N	Risk of bias (limitations of the included studies); Imprecision (the observed effect is small and the confidence interval almost reaches the no-effect line; confidence interval is wide); Publication bias	-
Zafer et al. 2016 [86]	Y	N	N	N	Risk of bias (Single-arm studies, no internal comparator）	-
Martins et al. 2016 [87]	Y	N	Y	N	Imprecision (observed as high heterogeneity (measured by the I2) and as changes in the direction of the observed effect); Indirectness (observed as high heterogeneity (measured by the I2) and as changes in the direction of the observed effect)	Plausible confounding (the influence of the most important confounding factor (maternal age) would reduce the observed effect)
Di et al. 2016 [47]	Y	N	Y	N	Risk of bias (lack of information about the allocation concealment methods (60% of the studies) and because the studies did not report either miscarriage rate or LBR); Imprecision (small sample size or very few studies included)	-

Park et al. 2016 [88]	Y	N	Y	N	Imprecision (optimal information sizes were not met or the 95% CI of the adjusted OR overlapped no effect and failed to exclude)	-
Barbosa et al.2016 [48]	Y	N	Y	N	Imprecision (there were a relatively low number of events and a wide confidence interval); Inconsistency (wide confidence interval)	-
Martins et al. 2016 [49]	Y	N	Y	N	Imprecision (the 95%CI was wide); Inconsistency (some larger studies observed no effect while others observed a benefit of the intervention); Publication bias (unexplainable)	-
Tsiami et al. 2016 [50]	Y	N	Y	N	Risk of bias (The proportion of information from studies at moderate/high risk of bias is sufficient to affect the interpretation of results); Indirectness (as the assessment for transitivity was limited by insufficient data, despite the fact that the baseline characteristics of the patients did not differ across studies); Imprecision (arising from the suboptimal information size and/or the presence of 95% CI including the unit); Publications bias (as we implemented an extensive search strategy, substantially lowering the likelihood for missed eligible studies)	-
Kollmann et al. 2016 [51]	Y	N	Y	N	Risk of bias; Publication bias (the plot was clearly asymmetric and the most precise studies suggest no effect while the less precise studies) Imprecision;	-

Wang et al. 2017 [89]	Y	N	Y	N	Inconsistency (The I square of sensitivity of two tests are >80%, P value < 0.001)	-
Armstrong et al. 2017 [103]	N	N	Y	Y (3 important)	Risk of bias (Outcome assessors not blinded in some case; Time interval between tests was sometimes lengthy); Imprecision (High heterogeneity amongst the RCTs that examine hysteroscopy in women with failed cycles of IVF with low numbers of participants and broad CIs. Single RCT that examines hysteroscopy in women undergoing their first cycle of IVF with a normal scan.); Inconsistency (Significant heterogeneity between studies); Indirectness (Studies involved women with pelvic pain or a history giving rise to a suspicion of pelvic pathology. Some studies did not involve subfertile/infertile women. PICO differed significantly between studies—some were women with known pre-existing pathology, others with none)	-
Berghella et al. 2017 [52]	Y	N	Y	N	Imprecision (studies included relatively few patients and few events and thus had wide CIs around the estimate of the effect and because the optimal information size was not reached); Indirectness (the different study design)	-
Berghella et al. 2017 [53]	Y	N	Y	N	Imprecision（Outcomes were imprecise because studies included relatively few patients and few events and thus had wide CIs around the estimate of the effect and because the optimal information size	-

					was not reached); Indirectness（because of the different interventions）	
Romero et al. 2017 [54]	Y	N	Y	N	Risk of bias (Most of the pooled effect provided by one study with moderate risk of bias); Imprecision (Small sample size and few events; 95% CI does not include effect)	-
Bechtejew et al. 2017 [55]	Y	N	Y	N	Risk of bias; Inconsistency (Although I^2 was very high, all studies have shown a large reduction on follicle-stimulating hormone consumption and number of oocytes); Imprecision	-
Nassr et al. 2017 [104]	Y	N	Y	N	Risk of bias; Indirectness; Imprecision (Downgrading reasons not explained)	-
Sjöström et al. 2017 [56]	Y	N	Y	N	Risk of bias (Overall unclear risk of bias in included studies); Imprecision (somewhat broad 95% CI that crosses the line of no effect); Inconsistency (Very high heterogeneity)	-
Meireles et al. 2017 [90]	Y	N	Y	Y (2 important,1critical)	Risk of bias (moderate according to Meta-Analysis of Statistics Assessment and Review Instrument); Inconsistency (I2> 50%)	Dose-response (Upgrading reason not explained)
Kim et al. 2017 [105]	N	N	Y	N	Risk of bias; Imprecision (Downgrading reasons not explained).	-
Pinto-Lopes et al. 2017 [57]	Y	N	Y	N	Risk of bias; Publication bias; Imprecision (Wide confidence interval crossing the line of no effect)	-

Saccone et al. 2017 [58]	Y	N	Y	N	Indirectness; Imprecision (Downgrading reasons not explained).	-
Berg et al. 2018 [101]	Y	N	Y	N	Imprecision (small sample size with imbalance between groups); Inconsistency (I2= 52%)	-
Grabovac et al. 2018 [106]	Y	N	Y	Y (7 critical)	Risk of bias (Risk of bias assessed as serious for all outcome since the average score for the Newcastle Ottawa Quality assessment scale was <6); Inconsistency (We assessed inconsistency as not serious for all outcomes); Imprecision (due to confidence intervals, which spanned no effect.); Publication bias (insufficient number of studies)	Large effect (A large effect/strong association was detected for death before discharge (23+0-27+6)
Maheshwari et al. 2018 [107]	Y	N	Y	N	not report	-
Luque-Ramírez et al. 2018 [59]	Y	N	N	N	Risk of bias; Imprecision; Inconsistency; Publication bias (Downgrading reasons not explained).	-
Cavoretto et al. 2018 [91]	Y	N	Y	Y (2 critical)	Risk of bias (5 out 15 studies did not control for maternal age and parity/all the 3observational studies considered in the sub-analysis controlled for maternal age, but 1 out of 3 did not control the party); Imprecision (95%CI includes OR of 1.0);	Plausible confounding (IVF group was more Risk of bias treated with progesterone

						compared to controls. Furthermore, IVF pregnancies are considered "precious pregnancies", thus fetal surveillance and maternal close attention to fetal health are generally more rigorous in IVF than in non-IVF pregnancies.
Alviggi et al. 2018 [92]	Y	N	N	N	Imprecision; Inconsistency; (Downgrading reasons not explained).	-
Senra et al. 2018 [60]	Y	N	Y	N	Risk of bias (All studies were open-label, which might have caused performance bias and/or detection bias); Indirectness (Some studies used amenorrhea as a surrogate for primary ovarian insufficiency, while others confirmed the diagnosis with elevated follicle-stimulating hormone levels);	-

					Imprecision (Seven studies estimated risk of pregnancy with large confidence intervals due to small number of events.)	
Kalafat et al. 2018 [61]	Y	N	Y	N	Risk of bias; Imprecision; Inconsistency; Indirectness (Downgrading reasons not explained).	-
Vitagliano et al. 2018 [62]	Y	N	Y	N	Risk of bias (Majority of studies at high/unclear risk of bias in four or more domains); Inconsistency (high heterogeneity among study populations, type of intervention and cointerventions); Imprecise (The number of participants is insufficient to detect a precise estimate of the effect)	-
Vitagliano et al. 2018 [63]	Y	N	Y	N	Risk of bias (risk of selection bias, risk of detection bias, risk of attrition bias, risk of reporting bias, risk of other bias); Inconsistency (heterogeneity in endometrial scratch injury techniques/timing and in ovarian stimulation protocols); Imprecision (small number of cases and events)	-
Siristatidis et al. 2018 [64]	Y	N	Y	N	Indirectness (embryological outcomes were primary endpoints); Imprecision (wide confidence intervals, reduced cohort size); Inconsistency (high heterogeneity)	-
Gadalla et al. 2018 [65]	Y	N	Y	N	Risk of bias (Most studies have unclear or high risk of selection, detection and performance bias); Inconsistency (There is substantial heterogeneity that was not improved in sensitivity analyses);	-

					Publication bias (Funnel plot for this outcome is almost symmetrical); Imprecision (total events < 300)	
Romero et al. 2018 [66]	Y	N	Y	N	Imprecise (95% CI does not include effect, lower and upper bounds <0.75 and >1.25; Small sample size)	-
Matthewman et al. 2018 [67]	Y	N	Y	Y (2 important)	Risk of bias (Most studies demonstrated high risk of bias in more than one area of study methodology); publication bias (Funnel plot asymmetry consistent with publication bias)	-
Fang et al. 2018 [68]	Y	N	Y	Y (2 important, 2 critical)	Risk of bias (Serious limitations in design); Inconsistency；Imprecision (Downgrading reasons not explained).	-
Vitagliano et al. 2019 [69]	Y	N	Y	N	Inconsistency (I2 = 39%, I2 = 42%); Imprecision (small number of events)	-
Jarde et al. 2019 [70]	Y	N	Y	N	Inconsistency (More than moderate heterogeneity was detected (I2 > 60%)); Indirectness (We estimated that there was intransitivity between the studies of the direct comparisons that underlie the indirect comparison)	-
Baiju et al. 2019 [71]	Y	N	Y	N	Imprecision (wide confidence intervals and small number of events); Inconsistency (heterogeneity is moderate)	-

Cai et al. 2019 [93]	Y	N	Y	Y (critical)	Risk of bias (The study subjects and the setting were not described in detail; invalid and unreliable tools were used to measure exposure and outcome variable. The criteria for inclusion in the sample was not clearly defined; invalid and unreliable tools were used to measure exposure variable.); Imprecision (The 95% CI crosses the line of no effect); Inconsistency (only one study and the heterogeneity was high (I2⩾50%))	-
Bosdou et al. 2019 [72]	Y	N	Y	N	Risk of bias (Most of the evidence is from studies with serious risk of bias); Inconsistency (Severe, non-explained, heterogeneity (I2⩾ 50%); Imprecision (wide confidence intervals)	-
Wang et al. 2019 [73]	Y	N	Y	N	Risk of bias; Inconsistency; Imprecision (Downgrading reasons not explained).	-
Haahr et al. 2019 [94]	Y	N	Y	N	Risk of bias; Inconsistency (Especially the significant effect on early spontaneous abortion speaks against the observed point estimate, I2 of 0%, I2 of 33%); Indirectness (populations were different in many ways although they were all IVF treated); Imprecision (as the 95%CI included clinically relevant effect estimates)	-

Saccone et al. 2019 [95]	Y	N	Y	Y (4 critical)	Risk of bias; Inconsistency (clinical and methodological heterogeneity among the included studies, small sample size of the included studies. Heterogeneity in the assessment of blood loss); Publication bias (large studies may have contributed more in the observed results. The allocation of different types of PAS severity in the different sub-groups may have also raised the systematic review findings)	Large effect; Plausible confounding
Sotiriadis et al. 2019 [74]	Y	N	Y	N	Risk of bias (data are derived exclusively from studies at concern for bias or at high risk of bias); Indirectness (Different direction of effect across studies); Imprecision (very small number of events)	-
Vermey et al. 2019 [96]	Y	N	Y	Y (9 important, 3 critical)	Risk of bias (Two studies did not provide adjustment for potential confounders either through regression analysis or through matched design, the exposed cohort is not a true representation of the community and the loss to follow up was not reported); Imprecision (wide confidence interval and small sample size); Inconsistency (Confidence intervals do not overlap, I2 is large and the null effect being that all studies have the same magnitude of effect is not statistically significant)	Large effect

Cai et al. 2020 [93]	Y	N	N	Y (59 critical)	Risk of bias (Invalid and unreliable tools; the reasons to loss to follow up was not described or explored; no strategy to address incomplete follow up utilized; The comparable of the groups was not clear; The cases and control were not matched appropriately; the confounders factors were not clearly identified; the strategies to deal with confounders factors were not clearly stated); Inconsistency(I2⩾50%); Imprecision (The 95% CI crosses the line of no effect)	-
Backes et al. 2020 [97]	Y	Y	Y	N	Risk of bias (Substantial variation in definitions of morbidities was noted, as was reporting of severity; Representative and defined population; although limited in number; Bayley Scales II and III are not equivalent assessments)；Imprecision(Wide pooled CI); Inconsistency(Substantial variation among point effect estimates; Wide CIs on many studies, others nonoverlapping; Similar results for sub-analyses based on cohort years, publication years, and center type; significant heterogeneity among studies; Some variation among point effect estimates on forest plot ; Overlap of CIs largely owing to the significant width of CIs among studies, particularly those with lower populations); Publication bias (significant asymmetry; Funnel plot shows all points inside “funnel”)	-

Li et al. 2020 [75]	Y	N	Y	N	Risk of bias; Imprecision; Inconsistency; Publication bias (Downgrading reasons not explained).	-
Qin et al. 2020 [76]	Y	N	Y	N	Risk of bias (Potential limitations); Imprecision (the sample size is small , total number of events <300); Indirectness (It's a secondary outcome which reduces confidence in the assessment of effect)	-
Danhof et al. 2020 [77]	Y	N	Y	N	risk of bias; imprecision; inconsistency (heterogeneity of results)	-
Bergeron et al. 2020 [43]	Y	N	Y	Y (3 critical，10 important)	Risk of bias (failure to follow intention to treat principles in analyses; Blinding of participants and personnel was not possible); Inconsistency (I^2 40% represents heterogeneity); Indirectness (included studies provide answers to the question；)	-
Zhang et al. 2020 [108]	Y	N	Y	Y (2 critical,4 not important,5 important)	Risk of bias; Imprecision; Inconsistency; Indirectness; Publication bias (Downgrading reasons not explained).	-
Islam et al. 2020 [78]	Y	N	Y	Y (1 critical，2 important)	Imprecision (confidence intervals included null value and too wide)； Inconsistency(I2=84%, p-value of heterogeneity (chi-squared)= 0.02 ; I2=63%, p-value of heterogeneity (chi-squared)= 0.07)；Publication bias (Some concern about small studies with negative effects)	-
Bordewijk et al. 2020 [79]	Y	N	Y	N	Risk of bias; Imprecision; Inconsistency (Downgrading reasons not explained).	-

Giorgione et al. 2020 [98]	Y	N	Y	N	Risk of bias (Undermatching in case-control studies; Failure to adequately control studies); Inconsistency (low p value)	Large effect; Plausible confounding
Varghese et al. 2020 [99]	Y	N	N	N	Risk of bias (Limitations in study design); Imprecision (small sample sizes and low number of events)	-
Bellos et al. 2020 [102]	Y	N	Y	N	Risk of bias (Owing to the retrospective design of the majority of studies that were included in the quantitative analysis); Imprecision (The small sample sizes resulted in wide confidence intervals, especially in the outcomes of severe-range hypertension and mean arterial pressure); Inconsistency (Severe hypertension and systolic and diastolic blood pressure, as the estimated I2values were high (>40%))	-
Cai et al. 2020 [84]	Y	N	Y	N	Risk of bias; Inconsistency (I2 > 50%, prediction intervals extend across the line of no effect); Imprecision (Sparse data, very sparse data,95% CI for RR includes 1.00)	-
Di Mascio et al. 2020 [81]	Y	N	N	N	Inconsistency; Indirectness (high heterogeneity within the included trials)	-
Joyeux et al. 2020 [100]	N	N	Y	N	Risk of bias (Limitations in study design and execution); Imprecision (small sample size <30 cases and small number of events, standard confidence interval); Indirectness (Lack of generalizability, transferability, applicability, external validity due to	-

					differences in populations, intervention, comparison and outcomes, heterogeneity of PICO criteria); Inconsistency (heterogeneity of results); Publication bias (systematic under- or over-estimate of effect due to selective publication).	
Samy et al. 2020 [82]	Y	N	Y	N	Risk of bias (Most of the studies did not report adequate methods of randomization or allocation concealment. Only 16 studies were double-blinded, whereas the rest of them were open-label or single-blinded trials); Imprecision	-
Shan et al. 2020 [109]	Y	N	Y	Y (17 critical, 7 important)	Risk of bias; Imprecision; Inconsistency; Indirectness (Downgrading reasons not explained).	-
Stewart et al. 2020 [83]	Y	N	N	Y (2 important)	Inconsistency (Only one trial was able to show non-inferiority); Imprecision (Wide confidence intervals)	-

CI, Confidence interval; N, No; NRSs, non-randomized studies; IVF, in vitro fertilization; RCTs, randomized controlled trials; Y, yes
